# PLGA/Gelatin/Hyaluronic Acid Fibrous Membrane Scaffold for Therapeutic Delivery of Adipose-Derived Stem Cells to Promote Wound Healing

**DOI:** 10.3390/biomedicines10112902

**Published:** 2022-11-11

**Authors:** Chia-Fen Hsieh, Chih-Hao Chen, Hao-Hsi Kao, Darshan Tagadur Govindaraju, Banendu Sunder Dash, Jyh-Ping Chen

**Affiliations:** 1 Department of Chemical and Materials Engineering, Chang Gung University, Kwei-San, Taoyuan 33302, Taiwan; 2 Department of Plastic and Reconstructive Surgery, Chang Gung Memorial Hospital at Keelung, Keelung 20401, Taiwan; 3 Department of Plastic and Reconstructive Surgery, Craniofacial Research Center, Chang Gung Memorial Hospital at Linkou, College of Medicine, Chang Gung University, Kwei-San, Taoyuan 33305, Taiwan; 4 Division of Nephrology, Chang Gung Memorial Hospital at Keelung, School of Medicine, College of Medicine, Chang Gung University, Kwei-San, Taoyuan 33302, Taiwan; 5 Department of Neurosurgery, Chang Gung Memorial Hospital at Linkou, Kwei-San, Taoyuan 33305, Taiwan; 6 Research Center for Food and Cosmetic Safety, College of Human Ecology, Chang Gung University of Science and Technology, Kwei-San, Taoyuan 33302, Taiwan; 7 Department of Materials Engineering, Ming Chi University of Technology, Tai-Shan, New Taipei City 24301, Taiwan

**Keywords:** wound healing, adipose-derived stem cells, hyaluronic acid, electrospinning, scaffold, cell delivery

## Abstract

Hyaluronic acid (HA) has been suggested to be a preferential material for the delivery of adipose-derived stem cells (ASCs) in wound healing. By incorporating HA in electrospun poly (lactide-co-glycolide) (PLGA)/gelatin (PG) fibrous membrane scaffolds (FMS), we aim to fabricate PLGA/gelatin/HA (PGH) FMS to provide a milieu for 3D culture and delivery of ASCs. The prepared FMS shows adequate cytocompatibility and is suitable for attachment and growth of ASCs. Compared with PG, the PGH offers an enhanced proliferation rate of ASCs, shows higher cell viability, and better maintains an ASC-like phenotype during in vitro cell culture. The ASCs in PGH also show upregulated expression of genes associated with angiogenesis and wound healing. From a rat full-thickness wound healing model, a wound treated with PGH/ASCs can accelerate the wound closure rate compared with wounds treated with PGH, alginate wound dressing, and gauze. From H&E and Masson’s trichrome staining, the PGH/ASC treatment can promote wound healing by increasing the epithelialization rate and forming well-organized dermis. This is supported by immunohistochemical staining of macrophages and α-smooth muscle actin, where early recruitment of macrophages, macrophage polarization, and angiogenesis was found due to the delivered ASCs. The content of type III collagen is also higher than type I collagen within the newly formed skin tissue, implying scarless wound healing. Taken together, using PGH FMS as a topical wound dressing material for the therapeutic delivery of ASCs, a wound treated with PGH/ASCs was shown to accelerate wound healing significantly in rats, through modulating immunoreaction, promoting angiogenesis, and reducing scar formation at the wound sites.

## 1. Introduction

Being a highly complex multicellular process, wound healing involves the coordination of different cell types and many cytokines [1]. The repair process can be divided into inflammation, tissue formation, and reconstruction stages, which involve interactions among dermal and epidermal cells, extracellular matrix (ECM), growth factors, and cytokines [2]. During the inflammatory stage, monocyte and neutrophil recruitment as well as macrophage activation occur [3]. The endothelial cells will migrate to the wound and proliferate in the next tissue formation stage where new blood vessels can be formed. At the same time, resident fibroblasts will form contractile granulation tissue by invading fibrin clots [4]. This process proceeds to form epidermal attachments after proliferation of epidermal stem cells. Therefore, a well-regulated coherent participation of many complex biological events occurs during healing of a cutaneous wound, which includes migration and proliferation of cells, angiogenesis, as well as ECM deposition and remodeling [5].

Stem cell-based therapy is gaining ground in skin wound management as the proliferation of stem cells and their signaling play a crucial role during every stage of the wound healing process, offering promise for the repair and/or replacement of damaged tissues and restoration of their lost functions [6]. In particular, cell therapy using adipose-derived stem cells (ASCs) has emerged as an attractive route to treat skin wounds due to their abundance, easy access, minimal invasiveness during, and large quantity after harvesting, as well as self-renewal and in vitro cell expansion capabilities [7]. It is widely known that ASCs can actively participate in different wound healing phases, through promoting the proliferation and migration of fibroblasts, accelerating neo-vascularization, secreting anti-inflammatory cytokines, regulating fibroblast phenotype, depositing ECM, and fastening re-epithelialization [8]. Furthermore, reduced scarring has recently been studied as one of the effects elicited by ASCs during skin regeneration. Although this effect may be related to the secretion of anti-scarring factors such as transforming growth factor-β3 (TGF-β3) by ASCs, the mechanism is still largely unknown [9].

Many studies employing locally transplanted ASCs to accelerate wound healing by taking advantage of their differentiation and vasculogenesis abilities in addition to the secretion of paracrine factors [10]. However, the ASCs after topical application will experience cell death within a short time, while retention of the transplanted cells in the delivery site is also severely limited [11]. To overcome the disadvantages associated with local delivery, a suitable biomaterial could be used as a delivery vehicle during transplantation of ASCs to maintain the cell viability. Hence, there is a urgent need for a cell delivery approach that can deliver ASCs to the wound site for recapitulating the complex microenvironments of ASCs and to maximize their therapeutic potentials [12]. The wound healing capacity from ASCs is synergistically regulated by soluble factors as well as by direct interaction with neighboring cells and ECM. Therefore, the local microenvironments in which ASCs reside plays a vital role in their phenotypic expression and determine their wound healing capacity [13]. To create a favorable microenvironment for ASCs to maximize their therapeutic potential, the properties of supporting biomaterial should be tailored to mediate the cell’s secretome [14]. The fibroblasts and keratinocytes can regenerate the wound tissue during the remodeling phase of wound healing by deposition of ECM. During wound healing, the delivered ASCs can differentiate into different cell types such as fibroblasts, keratinocytes, and endothelial cells, which may also secrete cytokines to promote their own proliferation and migration [15].

The tissue engineering approach for wound healing, where ASCs are combined with a biomaterial-based scaffold, is facing a major challenge to induce skin regeneration while avoiding scarring [16]. Recently, fibrous membrane scaffolds (FMS) prepared by electrospinning have attracted much attention for application in wound repair, which arises as their morphological and dimensional properties are similar to the ECM of native skin tissues [17]. Furthermore, by incorporating ECM-like molecules into an electrospun fibrous structure, a biomimetic microenvironment may be created to imitate the native environment for cell growth [18]. Therefore, it can be hypothesized that transplantation of ASCs, which are pre-cultured in FMS, may be a feasible approach for therapeutic delivery ASCs, pending the ability of the scaffold to support cell growth and properly maintain cell phenotype [19]. Undoubtedly, the use of FMS for ASCs culture could allow improved cellular migration, proliferation, and differentiation. Nonetheless, as the mechanical and structural properties of a scaffold will affect the function of delivered cells, careful development of FMS in ASC delivery is important.

Considering the materials for fabricating FMS for culture of ASCs, the biodegradable and biocompatible polymer poly (lactic-co-glycolic acid) (PLGA) has been approvable by the U.S. Food and Drug Administration (FDA) for clinical use as a wound dressing material. This polymer was reported to promote wound healing and accelerate angiogenesis by the sustained release of exogenous lactate [20]. Gelatin is formed from collagen after partial denaturation and degradation of collagen fibrils, and like collagen, it can attract fibroblasts during the wound healing process [21]. Due to its low cost and biodegradability, gelatin is widely used in wound management, which is further supported by other important characteristics such as its low antigenicity compared with collagen, and strong hemostatic effect [22]. Hyaluronic acid (HA) is a polysaccharide composed of D-N-acetylglucosamine and D-glucuronic acid and is endowed with unique characteristics for use in regenerative medicine [23]. As one of the main components of ECM, HA also exists in many biological fluids and participates in the processes of adhesion, migration, and proliferation of fibroblasts and keratinocytes, as well as in fetal wound healing [24]. It is involved in an array of biological functions in the human body, such as wound healing, inflammation, cell proliferation, migration, angiogenesis, etc. [25]. However, as gelatin and HA are both water-soluble, a gelatin and HA-containing FMS should be crosslinked for long-term biomedical applications.

To overcome the limitations of the available skin wound healing treatments, we proposed the use of FMS for the delivery of ASCs by offering a suitable environment to promote the secretion of growth factors and enhance ECM deposition with reduced scarring. We hypothesize that incorporating the skin ECM component HA in an electrospun PLGA/gelatin FMS could augment the phenotype of seeded ASCs by promoting vascularization and restoring the functional properties of wounded skin tissue. The purpose of this study was therefore to investigate the role of HA in a scaffold that is used for ASC delivery. We first demonstrate the successful preparation of PLGA/gelatin (PG) and PLGA/gelatin/hyaluronic (PGH) FMS for ASC culture. The advantages of incorporating HA in an FMS as a preferred cell delivery vehicle were studied by comparing the proliferation and phenotype of seeded ASCs to achieve the most effective cell carrier for wound healing. Finally, the impact of ASC-seeded PGH on wound healing was studied in a cutaneous excision model in rats to elucidate the functions of transplanted ASCs for accelerated wound healing.

## 2. Materials and Methods

### 2.1. Materials

Poly (lactic-co-glycolic acid) (PLGA) 50/50 (intrinsic viscosity = 0.45 dL/g, hydroxyl end groups) was provided by Green Chemical Inc. (Taipei, Taiwan). Hyaluronic acid (HA, average molecular weight = 1,300,000) was obtained from Bloomage Freda Biotechnology Corp., Ltd. (Jinan, China). Gelatin (from porcine skin, type A), polyethylene oxide (PEO) of 2,000,000 Da molecular weight, N-hydroxysuccinimide (NHS), and 1-(3-dimethylaminopropyl)-3-ethylcarbodiimide (EDC) were purchased from Sigma-Aldrich (St. Louis, MO, USA). Dulbecco’s modified eagle medium (DMEM), 2-(N-morpholino)ethanesulfonic acid, HyClone fetal bovine serum (FBS), ABsolute q-PCR SYBR Green Mix, and DAB Quanto Chromogen were purchased from Thermo Fisher Scientific (Waltham, MA, USA). The Live/Dead Viability/Cytotoxicity Kit for Mammalian Cells, 4′,6-diamidino-2-phenylindole (DAPI) for nuclear staining, and phalloidin-tetramethylrhodamine B isothiocyanate (phalloidin-TRITC) for F-actin staining were obtained from Life Technologies (Carlsbad, CA, USA).

### 2.2. Fabrication of PLGA/Gelatin (PG) and PLGA/Gelatin/HA (PGH) Fibrous Membrane Scaffolds (FMS)

For fabricating FMS, a 10 mL plastic syringe was filled with a polymer solution containing PLGA, gelatin, HA, or PEO prepared in formic acid. The syringe was placed in a KDS 100 syringe pump (KD Scientific, Holliston, MA, USA) for the delivery of polymer solution under a high electric voltage. A blunted stainless steel needle (23-gauge) was fitted to the syringe, which was connected with a high-voltage power supply provided by Glassman High Voltage (High Bridge, NJ, USA), and operated at 26 kV. By placing a collector 15 cm from the needle tip, the fibers were collected by an aluminum foil on the collector surface. For PG FMS, the polymer concentration is 21/11/0.5 (%, *w*/*v*) of PLGA/gelatin/PEO. For PGH FMS, the polymer concentration is 13/7/3/0.4 (%, *w*/*v*) of PLGA/gelatin/HA/PEO. The flow rate is set at 0.6 and 0.5 mL/h for PG and PGH, respectively. The FMS was removed from the aluminum foil and dried overnight under vacuum. For crosslinking, the scaffold was treated with 4% (*w*/*v*) EDC/NHS (5:1 weight ratio) in 99.5% ethanol for 24 h, and washed with copious distilled water before use.

### 2.3. Characterization of Electrospun Fibrous Membrane Scaffolds

A scanning electron microscope (SEM) (Hitachi S-3000N, Tokyo, Japan) operating at 15 kV was used to observe fiber morphology after sputter coating with Au for 30 s. The average fiber diameter was determined by randomly choosing 10 fibers each from 10 SEM images using ImageJ (Version 1.53t, NIH, Bethesda, MD, USA). The tensile mechanical properties of FMS were evaluated with an H1KT uniaxial tensile testing machine (Tinius Olsen, Salfords, UK). A 50 mm × 10 mm rectangular membrane was pre-immersed in phosphate-buffered saline (PBS) for 1 h and vertically mounted in the testing machine with two mechanical grippers, leaving a 30 mm gauge length for mechanical loading. The load deformation value was recorded using a 10 N load cell until reaching 30 mm elongation length at 5 mm/min crosshead speed. A stress–strain curve was obtained, from which the ultimate stress (tensile stress at break), ultimate strain (tensile strain at break), and Young’s modulus (slope within the linear region) could be determined. The thermal property of an FMS sample (10 g) was characterized using TGA Q50 from TA Instruments (New Castle, DE, USA) for thermogravimetric analysis (TGA) and derivative thermogravimetric analysis (DTA). The tested temperature was from room temperature to 750 °C at 10 °C/min heating rate under nitrogen.

### 2.4. In Vitro Cell Culture

The ASCs were isolated from the inguinal fat pad of Sprague Dawley (SD) rats (LASCO, Taipei, Taiwan), 12 weeks old and weighing ~250 g [26]. The cells were cultured in low-glucose DMEM supplemented with 10% FBS in a T-75 culture flask and incubated at 37 °C in a CO_2_ atmosphere containing 5% CO_2_. The culture medium was changed every 2–3 days until 90% confluence. The cells at passage 4 were used for the experiments. Then, 100 μL of ASCs was seeded onto a disk-shaped FMS (1.5 cm diameter) placed in a well of a 24-well culture plate at 1 × 10^5^ cell density. After cell attachment for 4 h, 1 mL of cell culture medium (low-glucose DMEM supplemented with 10% FBS and 1% penicillin/streptomycin) was added and cultured for 7 days. On days 1, 3, and 7, the morphology of ASCs was observed by SEM. For sample preparation, the FMS after cell seeding was rinsed in PBS, followed by 2.5% glutaraldehyde fixation for 2 h. A stepwise dehydration process using a 50% to 95% gradient ethanol concentration was used to dehydrate the sample, and the sample was finally immersed in absolute ethanol for 30 min and dried. After coating the sample with gold for 60 s, it was examined under SEM. The proliferation of ASCs was determined from the MTS assay with the CellTiter 96^®^ AQueous One Solution Cell Proliferation Assay Kit (Promega, Madison, WI, USA). The [3-(4,5-dimethylthiazol-2-yl)-5-(3-carboxymethoxyphenyl)-2(4-sulfophenyl)-2H-tetrazolium] salt in the MTS assay solution was reduced by dehydrogenase enzymes in metabolically active cells, and the formed purple formazan crystals were dissolved in dimethyl sulfoxide for solution absorbance measurements. The absorbance of the solution is measured at 492 nm (OD_492_), which is proportional to the number of live cells.

The viability of ASCs in FMS was determined using the Live/Dead Viability/Cytotoxicity Kit for Mammalian Cells (Thermo Fisher Scientific, Waltham, MA, USA). After being cultured for 7 days, the cell-seeded FMS was washed with PBS and incubated with 0.5 mL of staining solution at 37 °C for 30 min. The calcein-AM in the kit can stain live cells green from cellular esterase activity and the ethidium homodimer-1 can stain dead cells red from the loss of plasma membrane integrity. The live and dead cells were imaged under a confocal laser scanning microscope (Zeiss LSM 510 Meta, Jena, Germany) at excitation/emission wavelengths of 490/515 nm and 535/617 nm for live and dead cells, respectively. To assess the cytoskeletal structure of ASCs, a 7-day in vitro cultured FMS/ASCs sample was washed with PBS and fixed in 4% (*w*/*v*) paraformaldehyde at room temperature. The cells were permeabilized for 3 min with 0.1% Triton X-100, stained for 3 min with DAPI, and stained for 20 min with phalloidin-TRITC, before washing three times with PBS. The cytoskeletal arrangement was observed with a Zeiss LSM 510 Meta confocal laser scanning microscope. The nucleus shows blue fluorescence at 340/488 nm emission/emission wavelength after DAPI binding, and the F-actin microfilaments in the cytoskeleton show red fluorescence at 540/570 nm excitation/emission wavelength after phalloidin-TRITC binding.

For gene expression of ASCs in FMS after 7-day in vitro culture, quantitative real-time polymerase chain reaction (qRT-PCR) was used. After isolating total RNA with Trizol in DEPC-treated water, the RNA was reverse transcribed to complementary DNA (cDNA) with the QuantiTect Reverse Transcriptase Kit (Qiagen, Hilden, Germany). For internal control, glyceraldehyde-3-phosphate dehydrogenase (GAPDH) was used. Amplification was conducted for 50 cycles in a thermo cycler. Each cycle consisted of 15 min at 95 °C for denaturation, 15 s at 95 °C for annealing, and 1 min at 60 °C for extension. The RT-PCR reactions were carried out in a CFD-3120 detection system (Bio-Red, Hercules, CA, USA) and the SYBR Green RT-PCR kit was used to detect the products after PCR. The expression of each gene was evaluated in triplicate. The primers used were vascular endothelium growth factor (VEGF) (forward: 5′ TACCTCCACCATGCCAAGT 3′; reverse: 5′ TGCATTCACATTTGTTGTGC 3′), transforming growth factor-β1 (TGF-β1) (forward: 5′ GGCCGTACTGGCTCTTTACA 3′; reverse: 5′ TAGATTGGTTGCCGCTTTC 3′), TGF-β3 (forward: 5′ AAGAAGGAACACAGCCCTCA 3′; reverse: 5′ GCGGAAGCAGTAGTTGGTGT 3′), keratinocyte growth factor (KGF) (forward: 5′ CTGCCAAGTTTGCTCTACAG 3′; reverse: 5′ TCCAACTGCCAGGGTCCTGAT 3′) and GAPDH (forward: 5′ GCTTTGCCCCGCGATCTAATGTTC 3′; reverse: 5′ GCCAAATCCGTTCACTCCGACCTT.

### 2.5. Animal Studies

The wound model was created in twenty-week-old male SD rats (LASCO, Taipei, Taiwan). All animal protocols were approved by the Institutional Animal Care and Use Committee of Chang Gung University (IACUC approval no. CGU107-271, date of approval: 19 March 2019). By completely anesthetizing the animals with Zoletil-50 and Rompun through intraperitoneal injection, two full-thickness wounds of 2 cm × 2 cm dimensions were created on each side in the dorsum of each animal. The animals were randomly divided into 4 groups (*n* = 4, each group at each time point). The wound was covered with 2.5 cm × 2.5 cm gauze (control), commercial alginate dressing (Melgisorb Ag from Mölnlycke, Gothenburg, Sweden), PGH, or PGH/ASCs. The PGH/ASCs sample was prepared by seeding 3.5 × 10^5^ ASCs in 2.5 cm × 2.5 cm PGH and culturing for 7 days in vitro. The wounded area was covered with Tegaderm (3M*,* Saint Paul, MN, USA) and fixed with an elastic bandage to prevent the rat from removing the covering. The rats were kept in individual cages and observed daily throughout the experimental period. On days 3, 7, and 14 post-treatment, four rats were euthanized by CO_2_ inhalation and the dressing was removed. The wounds were grossly examined and digitally photographed with a constant optical zoom. To quantitatively determine the remaining wound size, each wound was traced with a transparent tracing paper along the epithelium-covered border of a wound. The tracing paper was scanned to analyze the wound size with the ImageJ software. The relative wound healing rate was calculated from the closure rate of the original wound (%) as B/A × 100, where A is the original wound area and B is the remaining wound area at a specific time.

The excised skin tissue retrieved from sacrificed animal was subject to histological analysis by immersing in 10% formaldehyde, dehydrating, and embedding in paraffin. A 5 μm thick tissue section was prepared for examination. The tissue slice was subject to hematoxylin–eosin (H&E) and Masson’s trichrome staining following standard protocols. For immunohistochemical (IHC) staining, nonspecific binding was blocked with Ultra V Block for 5 min, followed by incubation with diluted primary antibody for CD68, CD163, type 1 collagen (COL I), type III collagen (COL III), or α-smooth muscle actin (α-SMA) at 4 °C for 24 h. After rinsing, the slide was incubated with N-Histofine Simple Stain Rat MAX PO for 30 min, followed by color development with DAB Quanto Substrate. After hematoxylin counterstaining, the slide was examined under an IX-71 inverted optical microscope. For semi-quantitative analysis of the region of interest within the whole tissue area, the PAX-it image analysis software was used. For this analysis, five randomly chosen fields from the images were examined, from which the area percentages of COL I, COL III, α-SMA^+^ vessels, and the number of CD68^+^ and CD163^+^ macrophages were calculated.

### 2.6. Statistical Analysis

The results are reported as mean ± standard deviation (SD). Statistical differences were determined by one-way analysis of variance (ANOVA). The least significant difference (LSD) test was used, and the differences were considered statistically significant if *p* < 0.05.

## 3. Results

### 3.1. Preparation and Characterization of Fibrous Membrane Scaffolds (FMS)

**Figure 1:**
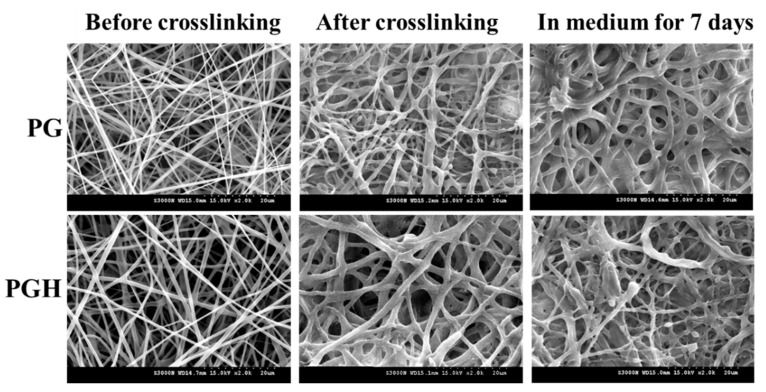
The scanning electron microscope (SEM) images of pristine and crosslinked PG and PGH fibrous membrane scaffold (FMS). The crosslinked FMS was immersed in cell culture medium for 7 days and examined by SEM for stability. Bar = 20 μm.

The scanning electron microscope (SEM) images of pristine FMS reveal a smooth fiber morphology of uniform fiber size (Figure 1). After crosslinking, the average fiber diameter increased from 664 ± 13 nm to 1014 ± 15 nm for PG and from 416 ± 12 nm to 716 ± 14 nm for PGH, with a moderate change in fiber morphology. Most importantly, the crosslinked FMS could maintain its fibrous structure during the in vitro cell culture period, as observed from the SEM images after immersing the FMS in cell culture medium for 7 days (Figure 1).

**Figure 2:**
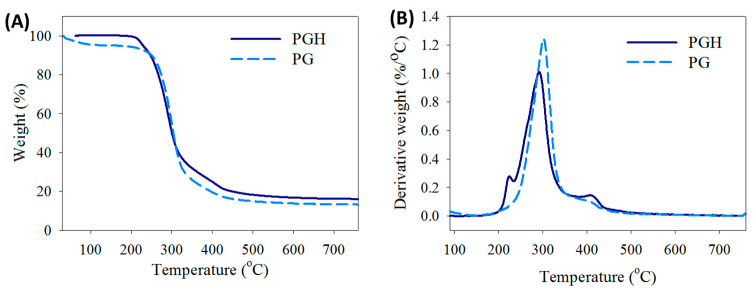
The thermogravimetric analysis (TGA) (**A**) and derivative thermograms (**B**) of PG and PGH fibrous membrane scaffold (FMS).

The thermal stability of the FMS was studied by thermogravimetric analysis (TGA) (Figure 2A). The PG shows a single decomposition peak at 300 °C due to the combined thermal decomposition of PLGA and gelatin. In contrast, the PGH shows a minor decomposition peak at 233 °C from HA in addition to a major decomposition peak at 290 °C (Figure 2B). Undoubtedly, the major decomposition peak arises from the decomposition of both PLGA and gelatin, which shifts slightly to a lower temperature due to interactions with HA. It should be noted that the residual weight of PGH (16.5%), after burning in nitrogen to 750 °C, is higher than that of PG (13.2%). This is due to the difference in residual weight between a natural polymer (HA or gelatin) (>0%) and a synthetic polymer (PLGA) (=0%) after complete thermal decomposition in nitrogen.

**Figure 3:**
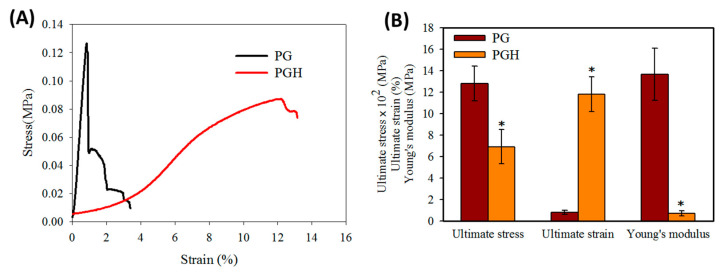
The tensile mechanical properties of PG and PGH fibrous membrane scaffold (FMS). (**A**) The typical stress–strain curves. (**B**) The ultimate stress, ultimate strain and Young’s modulus (*n* = 4). * *p* < 0.05 compared with PG.

The tensile strength of a membrane-type FMS is important during handling and application. The FMS should be endowed with sufficient mechanical properties, which can resist stretching during application [27]. For this purpose, FMS was subject to tensile mechanical testing, and typical stress vs. strain curves are shown in Figure 3A. The ultimate stress, ultimate strain, and Young’s modulus were determined from the stress vs. strain curves and compared in Figure 3B. Overall, the incorporation of HA significantly influenced the tensile strength of the FMS, as PGH shows significantly reduced Young’s modulus and ultimate stress. However, HA in PGH can substantially raise the ultimate strain 14.7-fold from PG, which is brittle and easily breaks at 0.8% strain.

### 3.2. In Vitro Cell Culture

**Figure 4:**
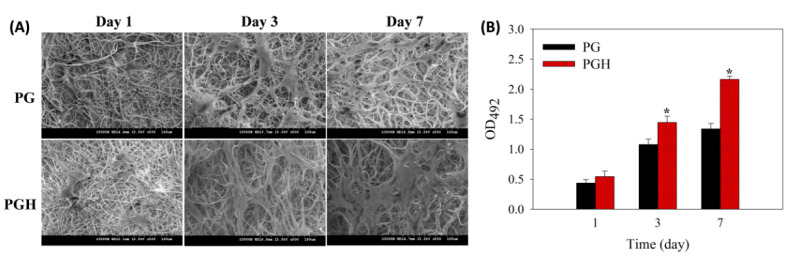
Scanning electron microscope (SEM) images (**A**, bar = 100 μm) and cell proliferation rate (**B**) after culture ASCs in PG or PGH scaffolds. The viable cell number was determined by the MTS assay by measuring the solution absorbance at 492 nm (OD_492_) in (**B**). * *p* < 0.05 compared with PG.

In order to investigate whether the FMS could support attachment, proliferation, and maintenance of the phenotypic profile of ASCs, we seeded ASCs in FMS and cultured for 7 days in vitro. The morphological appearance of cell-seeded FMS was examined from time-lapsed SEM images to confirm cell migration and proliferation. After cell seeding, a mesenchymal stem cell-like phenotype was observed from attached ASCs, which can maintain the undifferentiated spindle-like morphology in normal cell culture medium with protruded lamellipodia attaching firmly to the filamentous fibers (Figure 4A). The PGH was more rapidly colonized by ASCs than PG within 7 days. Furthermore, the ASCs in PG incline towards altered cell morphology on day 7, where a flat cell morphology with fibroblast-like appearance was observed. The proliferation of ASCs in FMS was evaluated quantitatively by using the MTS assay for the determination of viable cells in the scaffold. As shown in Figure 4B, the cell attachment rate showed no significant difference between scaffolds on day 1. However, the viable cell number was significantly higher in PGH on days 3 and 7. The increase in OD_492_ from day 1 to day 7 was 2.9-fold and 4.0-fold for PG and PGH, respectively, which indicates that PGH may provide a better milieu for the proliferation of ASCs.

**Figure 5:**
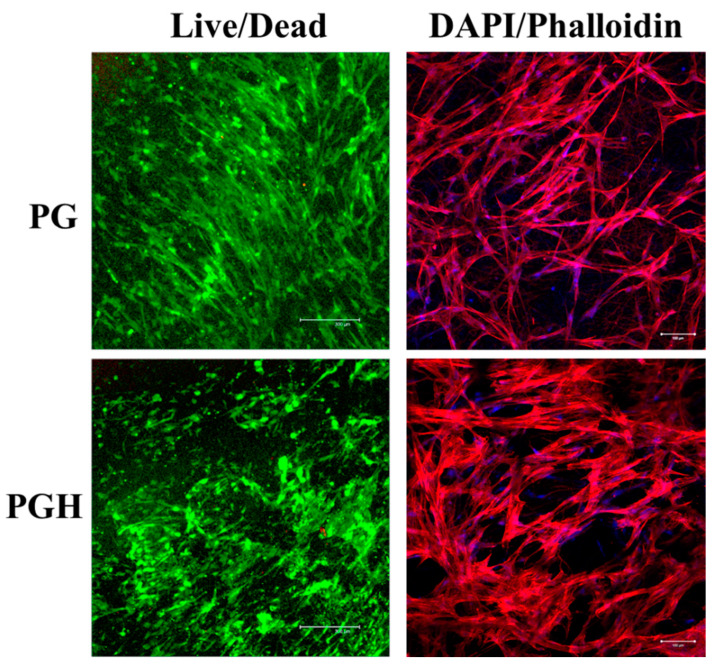
The confocal microcopy images after culture ASCs in PG and PGH fibrous membrane scaffolds (FMS) for 7 days. The merged images from Live/Dead staining show live cells in green and dead cells in red (bar = 300 μm). The merged images from DAPI/phalloidin staining show actin cytoskeleton in red and cell nuclei in blue (bar = 100 μm).

The confocal microscopy analysis was used to elucidate cell viability from Live/Dead staining, and cell morphology was examined from cytoskeleton staining on day 7 (Figure 5). As expected, the ASCs reveal high cell viability when cultured in both FMS. Only a few dead cells showing red fluorescence but abundant viable cells showing green fluorescence could be found. However, more dead cells and less live cells were found in PG, which is consistent with the number of viable cells determined from the MTS assay (Figure 4B). The ASCs in FMS were stained with rhodamine-labeled phalloidin for cytoskeleton arrangement and with DAPI for nuclei (Figure 5). The red fluorescence due to actin microfilaments in ASCs was evident in both scaffolds on day 7, albeit more elongated stress fibers composed of actin filament bundles were found in PG.

**Figure 6:**
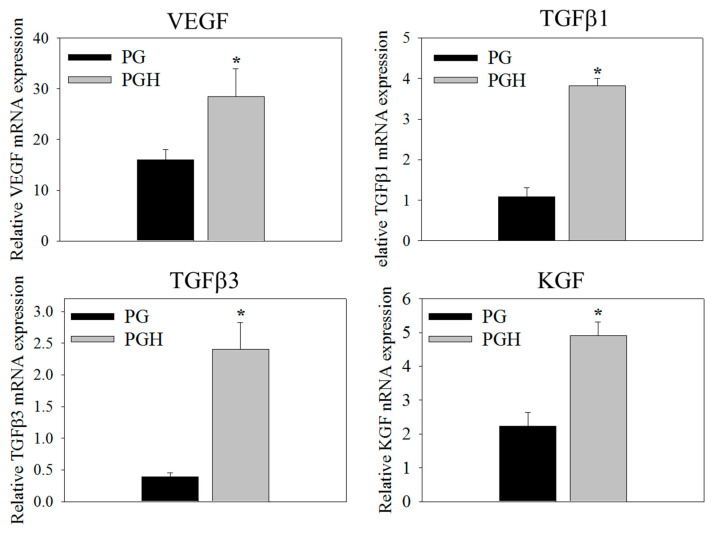
The gene expression analysis of ASCs cultured in PG or PGH FMS for 7 days in vitro. The relative mRNA of vascular endothelium growth factor (VEGF), transforming growth factor-β1 (TGF-β1), transforming growth factor-β3 (TGF-β3), and keratinocyte growth factor (KGF) was analyzed by quantitative real-time polymerase chain reaction (qRT-PCR). * *p* < 0.05 compared with PG.

To study whether HA can stimulate the expression of genes important for skin regeneration, relative mRNA expressions of VEGF, TGF-β1, TGF-β3, and KGF were determined by qRT-PCR on day 7. Compared to cells in PG, ASCs in PGH show significantly up-regulated gene expression for all tested genes (Figure 6).

### 3.3. Animal Studies

**Figure 7:**
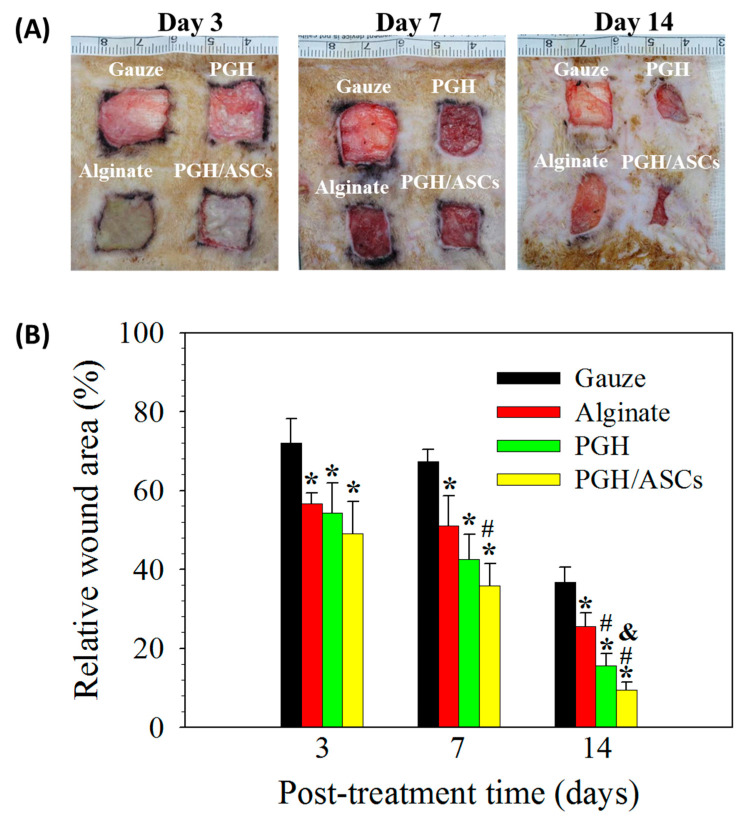
(**A**) The representative gross view images of wounds in SD rats on days 3, 7, and 14 after treatment with gauze, alginate wound dressing, PGH, or PGH/ASCs. (**B**) The relative wound area for each treatment was determined from the traced wound border covered with epithelia and normalized to the initial wound area on day 0 (*n* = 4). * *p* < 0.05 compared with gauze, ^#^ *p* < 0.05 compared with alginate, ^&^ *p* < 0.05 compared with PGH.

The wound healing ability was determined from a rodent critical-sized wound model created in SD rats. The wound size change was evaluated by covering the wound with gauze, alginate wound dressing, PGH, or PGH/ASCs. The dressing was removed from the wound on day 3, 7, or 14 in each group and the wound was grossly observed for wound healing (Figure 7A). The relative wound area was calculated by normalizing the remaining wound area at a specific time with its initial wound area on day 0. The wound closure rate was compared among groups treated with gauze, alginate, PGH (without ASCs), or PGH/ASCs (PGH seeded with ASCs and cultured in vitro for 7 days). The wounds treated with PGH and PGH/ASCs showed an accelerated wound healing rate, and the PGH/ASC treatment provided the best overall wound healing outcomes. As shown in Figure 7B, as early as 3 days post-treatment, all groups showed significantly decreased relative wound area when compared with gauze. All treatments continuously accelerated wound closure with time from day 7, and a significant difference in relative wound area was found when compared with gauze. However, the PGH/ASCs group started to show a significant difference from the alginate group at this time point. On day 14, the accelerated wound healing ability provided by PGH/ASCs manifests itself by significantly reducing the relative wound area compared with all other groups. Indeed, the relative wound area is in the order of PCH/ASCs (9.4 ± 2.1%) < PGH (15.6 ± 3.0%) < alginate (25.5 ± 3.4%) < gauze (36.7 ± 3.9%).

**Figure 8:**
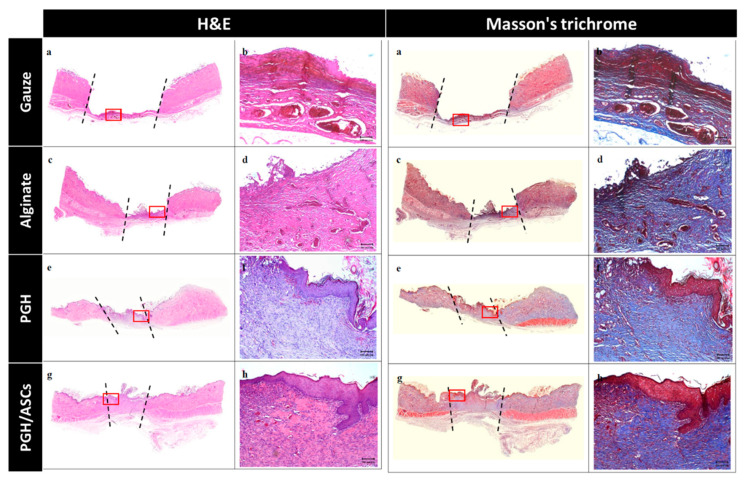
The histological analysis of wounds after treatment with gauze (**a**,**b**), alginate wound dressing (**c**,**d**), PGH (**e**,**f**), or PGH/ASCs (**g**,**h**) for 14 days by hematoxylin and eosin (H&E) and Masson’s trichrome staining (bar = 100 μm). A low-power image of each wound is shown to the left, with dotted lines indicating the boundary of created wounds. A high-power image taken from the red rectangle in the low-power image is shown to the right.

The skin tissue samples harvested from each group were examined by H&E and Masson’s trichrome staining on day 14. From H&E stains, the difference in re-epithelialization rate and granulation tissue formation is clearly demonstrated. Specifically, differentiated epithelia in the epidermis layers of PGH/ASC-treated wounds were found. This is different from a less-differentiated epidermis in PGH-treated wounds or incomplete epithelization accompanied by fibrinous debris in gauze or alginate-treated wounds (Figure 8). The PGH and PGH/ASCs groups also demonstrate different degrees of migration of the epithelium over the dermis and granulation tissue formation, in contrast to poor epithelium formation in the control and alginate groups. However, the PGH/ASC treatment led to complete re-epithelialization of the wound and significantly increased deposition of connective tissue. This treatment is also associated with a more stratified epidermal layer showing clear epidermal–dermal junctions. In Masson’s trichrome staining images, the red color indicates muscle fiber and keratin. The blue color indicates stained collagen, which can be used to reveal collagen deposition and collagen fiber alignment. Masson’s trichrome staining shows distinct collagen structures in the dermal layer of the PGH/ASCs group with increased collagen synthesis and neovascular structure, as visualized from H&E staining images, as well. These newly formed vessels can promote vascularization and accelerate wound healing.

**Figure 9:**
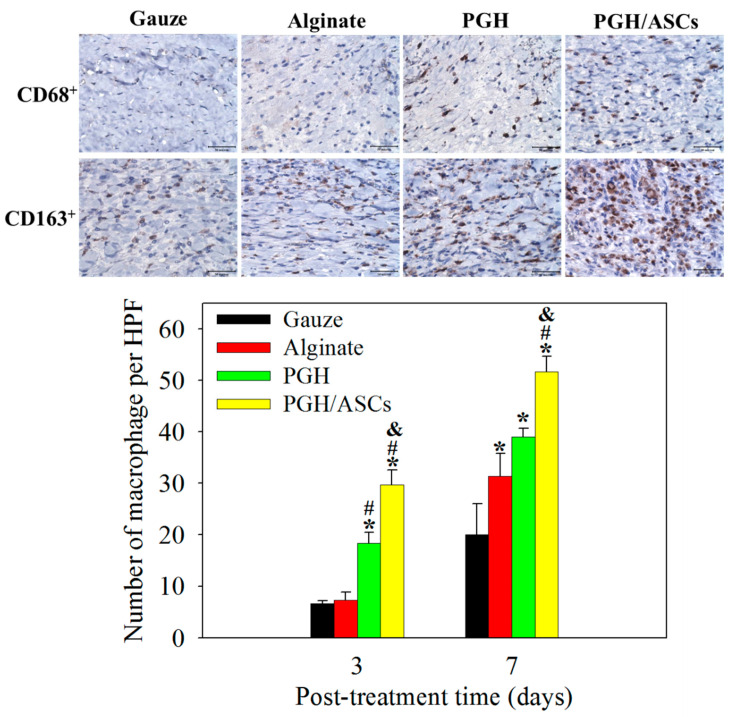
The representative immunohistochemical (IHC) staining images of CD68^+^ macrophage (day 3) and CD163^+^ macrophage (day 7) in wound tissues of SD rats after treatment with gauze, alginate wound dressing, PGH, or PCH/ASCs (bar = 50 μm), and the number of macrophages per high-power field (HPF) (*n* = 4). * *p* < 0.05 compared with gauze; ^#^ *p* < 0.05 compared with alginate; ^&^ *p* < 0.05 compared with GPH.

The expression of CD68, a marker for macrophages, was detected by immunohistochemical (IHC) staining. The number of CD68^+^ macrophages was significantly increased in the PGH group 3 days post-treatment (Figure 9). Moreover, the CD163^+^ macrophage population showed a significant increase in the PGH/ASCs group when compared with all other groups on day 7, indicating the increased population of alternatively activated M2 macrophages. At this time point, although both material-based treatment groups (alginate and PGH) show significant differences in CD163^+^ macrophages from the gauze group, no significant difference was found between them. The in vivo experiments thus demonstrate that ASCs delivered by PGH may exhibit an immunomodulatory effect by early recruitment of macrophages to the wound bed, followed by increasing the number of M2 macrophages by polarization.

**Figure 10:**
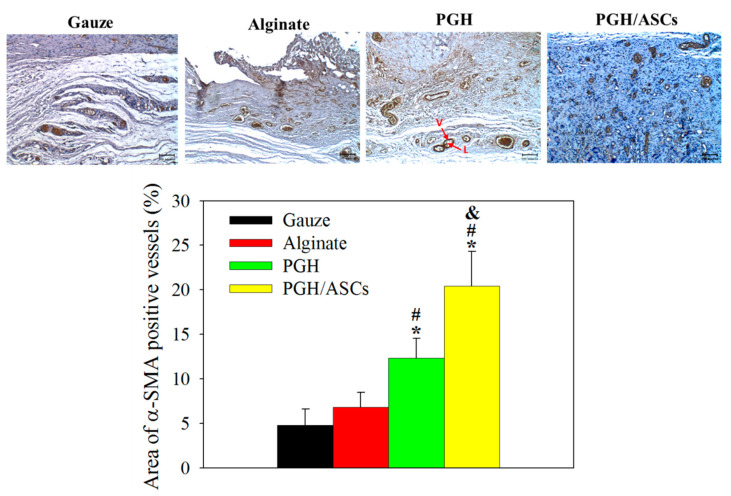
The representative immunohistochemical (IHC) staining images of α-smooth muscle actin (α-SMA) (bar = 100 μm; V, vessel; L, lumen) in wound tissues of SD rats after treatment with gauze, alginate wound dressing, PGH, or PCH/ASCs for 14 days, and the area percentage of α-SMA-positive vessels (*n* = 4). * *p* < 0.05 compared with gauze; ^#^ *p* < 0.05 compared with alginate; ^&^ *p* < 0.05 compared with PGH.

Increased α-SMA expression by myofibroblasts and pericytes is shown from the wound treated with PGH/ASCs on day 14 from IHC staining (Figure 10). This staining result for the PGH/ASCs group indicates that the vascularization of wounds is not present in other groups. Indeed, abundant blood vessel formation from stained pericytes in blood vessel walls, which secrete positively stained α-SMA marker protein, was found after PGH/ASC treatment (Figure 10). Follow-up semi-quantitative image analysis shows a significantly higher area percentage of α-SMA-positive vessels in the PGH/ASCs group when compared with other groups (Figure 10).

**Figure 11:**
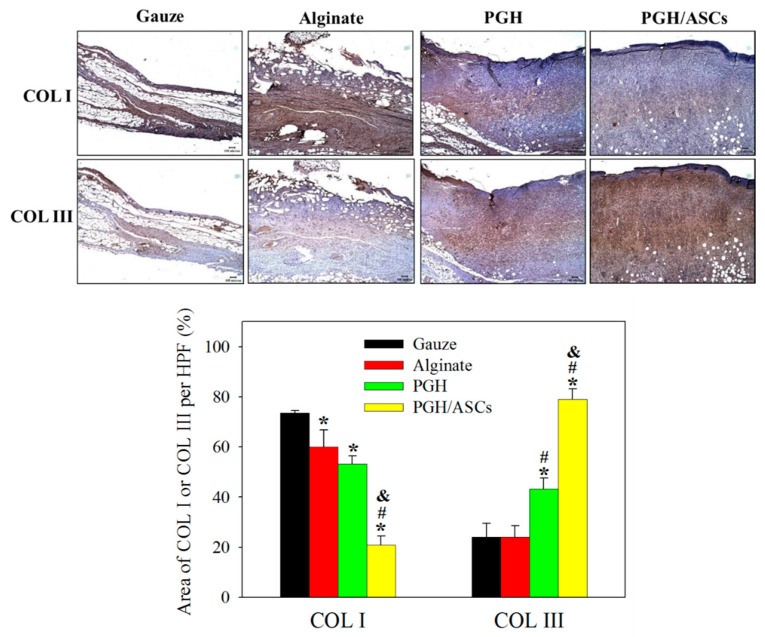
The representative immunohistochemical (IHC) staining images of type 1 collagen (COL I) and type 3 collagen (COL III) (bar = 100 μm) in wound tissues of SD rats after treatment with gauze, alginate wound dressing, PGH, or PCH/ASCs for 14 days, and the area percentage of COL I and COL III per high-power field (HPF) (*n* = 4). * *p* < 0.05 compared with gauze; ^#^ *p* < 0.05 compared with alginate; ^&^ *p* < 0.05 compared with PGH.

To investigate possible differences in scar formation, the IHC staining of COL I and COL III in treated wounds was conducted (Figure 11). The area percentage expressing COL I or COL III within each high-power field was then estimated from the IHC images. As the area percentage is calculated within the tissue, the different tissue thickness in Figure 11 will not influence the comparison between treatments. The wound covered with PGH/ASCs shows the lowest area percentage of COL I (20.9 ± 3.6%) and the highest area percentage of COL III (79.0 ± 4.1%), which are significantly different from all other groups, implying minimum scar tissue formation (Figure 11). Indeed, the average area ratios of COL I/COL III were calculated to be 3.1, 2.5, 1.2, and 0.3 for gauze, alginate, PGH, and PGH/ASCs, respectively.

## 4. Discussion

Although the use of FMS for ASC delivery can lead to improved cell migration, proliferation, and differentiation, the physico-chemical properties of these scaffolds demand careful analysis [28]. From SEM analysis, the crosslinking treatment is deemed necessary when FMS is intended for the delivery of ASCs, as the scaffold can maintain its stability throughout the in vitro culture period (Figure 1). The crosslinked FMS was thus employed exclusively in this study. The successful incorporation of HA in PGH could be concluded from TGA analysis, which shows a decomposition peak temperature associated with HA (Figure 2). Furthermore, the PGH shows higher residual weight than PG after complete thermal decomposition in nitrogen, which reflects a higher mass percentage of PLGA in PG (66%) than PGH (57%), as calculated from the composition of the spinning solutions. This is due to the nature of components in the FMS, as PLGA is a synthetic polymer providing 0% residual weight in contrast to a natural polymer such as HA or gelatin providing some residual weight [29]. From mechanical testing, the tensile mechanical strength of both scaffolds is within an acceptable range to cover a wound with a membrane-type scaffold, which is also suitable for loading and delivery of ACSs (Figure 3) [30]. However, the increased ultimate strain associated with PGH may provide higher flexibility to withstand higher stretching during its application to the wound.

The better maintenance of cell morphology supports the preferential use of PGH for ASC culture (Figure 4A), as previous reports indicate that changes in the morphology of ASCs following in vitro expansion can reduce their proliferation potential [31]. This is consistent with a higher cell proliferation rate for ASCs cultured in PGH, which indicates that this FMS can provide a better milieu for the growth of ASCs (Figure 4B). Taken together with the confocal microscopy analysis results in Figure 5, ASCs were found to proliferate and survive better while preserving their phenotype in PGH. Furthermore, all target genes associated with skin regeneration were upregulated in PGH, which may increase the growth factor secretion by ASCs to collectively enhance their wound healing functions (Figure 6) [32]. The mechanisms of wound healing associated with ASCs are complex and diverse and they may participate in the entire wound healing process, including inflammation, proliferation, and remodeling [33]. During the inflammation stage, the ASCs can induce transformation of macrophages from pro-inflammatory M1 to anti-inflammatory M2 phenotype to regulate inflammation [34]. During the proliferation and remodeling phase, the ASCs can secrete growth factors such as VEGF, TGF-β, insulin growth factor (IGF), hepatocyte growth factor (HGF), and platelet-derived growth factor (PDGF). These biological factors can promote the proliferation and migration of fibroblasts, the synthesis of collagen and other ECM components, and the growth of new blood vessels during wound healing [35,36]. Considering growth factors involved in wound healing and skin regeneration, the VEGF can stimulate angiogenesis as well as enhance endothelial cell migration and proliferation [37]. All isoforms in the TGF-β family were reported to be positively correlated with accelerated wound healing [38], with TGF-β1, TGF-β2, and TGF-β3 showing overlapped functions [39]. Both TGF-β1 and TGF-β3 act as a potent chemo-attractant for macrophages and as a mitogen for fibroblasts [40]. They also promote the formation of granulation tissue and regulate the tensile strength of neo-skin tissue [41]. However, some research suggests that TGF-β1 may be related to fibrotic scar formation, which is different from the TGF-β3-induced scarless wound healing. Nonetheless, the underlining mechanism responsible for such a difference appears to be far more complex. For KGF, it plays an important role during the repair of injured epithelium and the induction of activin in granulation tissue formation [42].

Overall, from the assessment of the relative wound area (Figure 7) and from histological analysis (Figure 8), the PGH/ASC treatment can significantly accelerate wound healing by regenerating skin with a defined underlying collagen layer in 2 weeks compared to wound healing in other groups. During wound healing, the neutrophils will migrate to the wound bed to initiate the initial inflammatory response to repair the wound. This is followed by the movement of macrophages into the sites to secrete cytokines, and the secreted cytokines can attract cells responsible for wound repair [43]. As a reduction in macrophage infiltration is associated with significantly delayed wound healing, the delivery of macrophage-activating ASCs into healing wounds is expected to augment its repair process. By participating in ECM remodeling and granulation tissue formation during wound repair, macrophages are critical for proper wound healing [44]. However, the macrophages will undergo phenotypic change during the wound healing process. By changing from activated pro-inflammatory M1 to alternatively activated anti-inflammatory M2 phenotype, such a transition contributes to changing a pro-inflammatory to a pro-resolution wound microenvironment [45]. Upon initial infiltration, the M1 macrophages will remove damaged matrix, cellular debris, and neutrophils. They begin to transition into an M2 state during new tissue formation by secreting anti-inflammatory cytokines to promote ECM synthesis and wound contraction. These anti-inflammatory macrophages help the reorganization of the ECM along tension lines with concurrent phagocytosis of remaining debris during the final wound healing stage [46]. Moreover, the anti-inflammatory M2 macrophages play an important role in angiogenesis. Other than degrading ECM to create tunnels and guide endothelial proliferation and migration, M2 macrophages also release angiogenic factors [47]. Therefore, the early recruitment of macrophages to the initial inflammatory response is instrumental for supplying the biological signals to direct cell movement, which is necessary for wound healing. As shown in Figure 9, the number of CD68^+^ macrophages significantly increased after 3 days of treatment with PGH/ASCs. This significantly increased M1 macrophage population in the wound area endorses the ability of delivered ASCs to recruit macrophages early during the inflammation phase of wound healing. Furthermore, the marked increase in the CD163^+^ macrophage population in the PGH/ASCs group on day 7 indicates its higher macrophage polarization ability for transforming into the anti-inflammatory M2 phenotype [48]. The ASCs delivered by PGH can therefore recruit macrophages to the wound bed and induce non-activated M1 macrophages into anti-inflammatory regulatory M2 macrophages to promote healing and inhibit inflammation/immune response [49]. Overall, the results shown in Figure 9 are consistent with the critical role that macrophages play in ASC-mediated wound healing, indicating that PGH/ASC treatment can deliver ASCs and enhance the recruitment of macrophages to the wound bed, as well as increase the M2 macrophage population by inducing macrophage polarization towards the M2 phenotype [50].

Both neovascularization and angiogenesis represent important considerations of wound healing outcomes, as the newly formed blood vessels can provide nutrition and oxygen to growing tissues and promote wound healing [51]. Thus, a higher angiogenesis rate in the wound bed can enhance the dermal layer formation rate. As a marker of myofibroblasts, the α-smooth muscle actin (α-SMA) is largely responsible for wound contraction and ECM production [52]. Other than myofibroblasts, pericytes also synthesize α-SMA, which wrap around the capillary vessel after blood vessel maturation [53]. A higher extent of α-SMA marker expression can thus contribute to blood vessel maturation and wound contraction. The roles ASCs play in promoting wound healing have been linked to their ability to promote angiogenesis [54]. By topically administering ASCs to ulcers in diabetic mice, enhanced wound closure by neovascularization was previously shown [55]. Enhanced wound healing in mice has been found from the differentiation of ASCs into endothelial cells [56]. Overall, the enhanced expressions of α-SMA, a widely used marker for blood vessel formation, supports that PGH/ASCs can promote endothelial cell differentiation and accelerate blood vessel maturation to meet the demand of oxygen and nutrients during wound repair (Figure 10) [57]. Other than angiogenesis, we also found vast enhancement of COL III over COL I expression in the neo-skin tissue of PGH/ASC-treated wounds (Figure 11). It has been shown that collagen subtype deposition can predict scar formation, as fetal skin is known to contain a greater proportion of COL III in comparison with COL I [58]. This differential collagen deposition is thought to contribute to scarless wound healing [59]. As fetal skin contains a higher proportion of COL III in contrast to adult skin that consists mostly of COL I, the types of collagen in adult skin show an increased COL I/COL III ratio as the fetus develops, which also correlates with a shift from scarless wound healing to scar formation [60]. Indeed, histologic examinations in healed fetal wound tissue demonstrate a higher COL III/COL I ratio than adults [61]. Taken together, this implies that the COL III/COL I ratio may be a useful target to assess wound scaring [62]. Furthermore, a hypertrophic scar is usually associated with excess deposition of COL I [63]. Although considering COL III/COL I ratio alone for scarless wound healing may be limited by the complex nature of the factors involved, the histological assessment of COL I and COL III remains a possible way to measure outcomes in scarring research [64]. Overall, our findings suggest that skin wound healing is accelerated and less prone to scar formation in the presence of PGH/ADSCs, which may be related to a controlled inflammatory process associated with a high level of alternatively activated macrophages, as well as linked to increased angiogenesis in wound areas.

## 5. Conclusions

We demonstrate that PGH is a preferred cell carrier for ASC delivery in cellular regenerative therapy to accelerate wound healing. The PGH FMS can accommodate ASCs and be used as a vehicle for therapeutic cell delivery. Incorporating HA can produce a PGH FMS that can enhance the wound healing function of ASCs by increasing the cell proliferation rate and upregulating the gene expression level of maker genes important for wound healing, as well as better maintain cell phenotype. By accommodating the survival and proliferation of ASCs, followed by the delivery ASCs to the wound site, the PGH/ASC treatment is shown to modulate inflammation through the recruitment and polarization of macrophages, as well as to promote angiogenesis for possible scarless wound healing.

## Data Availability

The data presented in this study are available on request from the corresponding author.
